# Critical Kennedy’s disease with Guillain-Barré syndrome and respiratory failure: A case report

**DOI:** 10.1097/MD.0000000000050722

**Published:** 2026-09-25

**Authors:** Xinran Gao, Kunyao Yu

**Affiliations:** a Department of Respiratory and Critical Care Medicine, Peking University First Hospital, Beijing, China.

**Keywords:** Guillain-Barré syndrome, Kennedy’s disease, respiratory failure, SBMA, spinal and bulbar muscular atroph

## Abstract

**Rationale:**

Spinal and bulbar muscular atrophy (SBMA, Kennedy disease) is a rare X-linked progressive neurodegenerative disorder. Evidence on the intensive care management of severe SBMA with acute decompensation remains scarce, and superimposed treatable immune-mediated neuropathies are often overlooked in such clinical scenarios.

**Patient concerns:**

A 61-year-old man with a 35-year history of progressive limb weakness and 3 months of worsening dysphagia was transferred to our hospital for acute respiratory failure. He had developed progressive dyspnea, ineffective cough, and secretion retention over 15 days, and had been intubated for hypoxemic respiratory failure at the referring facility.

**Diagnoses:**

Genetic testing confirmed SBMA as the underlying diagnosis, accounting for the patient’s chronic progressive weakness, bulbar dysfunction, and chronic neurogenic electrophysiologic changes. Superimposed probable Guillain–Barré syndrome was suspected based on acute monophasic deterioration, generalized areflexia, sensorimotor axonal neuropathy, and positive anti-ganglioside antibodies, though diagnostic certainty was limited by normal cerebrospinal fluid protein and confounding chronic SBMA pathology. Concurrent conditions included pneumonia, acute segmental pulmonary embolism, and acute respiratory failure.

**Interventions:**

Multidisciplinary management was delivered, including invasive respiratory support, targeted antimicrobial therapy, therapeutic anticoagulation, aggressive bronchoscopic airway clearance, and structured pulmonary and neuromuscular rehabilitation. Four cycles of therapeutic plasma exchange were administered given ongoing suspicion of immune-mediated polyradiculoneuropathy.

**Outcomes:**

Oxygenation and muscle strength improved gradually with combined interventions, and no single treatment could be identified as the sole contributor. The patient experienced extubation failure on hospital day 18 due to persistent airway protection deficits, and underwent tracheostomy on day 26. He was successfully weaned from invasive ventilation and discharged to a rehabilitation facility on hospital day 44.

**Lessons:**

Acute neurological deterioration in patients with SBMA should prompt evaluation for superimposed, potentially treatable neuromuscular disorders, rather than being attributed exclusively to baseline disease progression. Integrated multidisciplinary care is essential to optimize outcomes in severe SBMA complicated by acute respiratory decompensation.

## 1. Introduction

Spinal and bulbar muscular atrophy (SBMA), also known as Kennedy’s disease, is a rare X-linked, progressive neurodegenerative disorder.^[1]^ It is caused by the expansion of a CAG repeat in the first exon of the gene encoding the androgen receptor, which is located on the X chromosome.^[2]^ The estimated prevalence of SBMA ranges from 1 to 2 per 100,000 individuals, primarily due to underdiagnosis.^[3]^ Recent epidemiological data from Italy reported a prevalence of 2.58 per 100,000 individuals in the male population.^[4]^ The initial symptom of SBMA is typically hand tremor.^[5]^ Other symptoms may include gynecomastia, orofacial fasciculation, limb weakness, dysarthria and dysphagia.^[5]^ A clinical survey conducted among 61 patients with Kennedy’s disease in the UK identified lower limb muscle weakness as the most common manifestation (86.7%), whereas respiratory involvement was observed in only 5% of cases.^[6]^ A 20-year follow-up study of 223 Japanese patients with Kennedy’s disease revealed that pneumonia secondary to dysphagia or aspiration was the primary cause of death among 15 deceased patients.^[7]^ Herein, we report a case of genetically confirmed SBMA presenting with acute respiratory failure, in whom superimposed probable Guillain–Barré syndrome (GBS) was suspected and comprehensively managed with a multidisciplinary strategy.

## 2. Case presentation

A 61-year-old man was transferred to our hospital with acute respiratory failure after 15 days of progressive dyspnea. He had developed symmetric lower-extremity weakness and numbness at age 26 years. Lumbar discectomy 2 years later provided little benefit, and his gait progressively deteriorated. Upper-extremity weakness began at age 54 years. Three months before admission, he developed progressive dysphagia and lethargy. During the 2 weeks before transfer, dyspnea, ineffective cough, secretion retention, and impaired consciousness developed. He was intubated for acute hypoxemic respiratory failure at the referring hospital. His parents had no similar symptoms. One brother reported limb weakness but had not undergone neurologic or genetic evaluation. No neuromuscular symptoms were reported among the patient’s offspring.

On admission, the patient was receiving invasive mechanical ventilation and was hemodynamically stable. Examination showed bilateral chemosis and coarse breath sounds. Neurologic examination revealed symmetric weakness of the facial, axial, neck flexor, and limb muscles, with prominent temporal and distal limb atrophy. Muscle tone was reduced, and deep tendon reflexes were absent throughout. Despite the remote history of lower-extremity numbness, no objective sensory loss was identified. No clinically apparent autonomic dysfunction was documented.

Admission laboratory findings are summarized in Table 1. CT pulmonary angiography showed acute segmental pulmonary embolism in the right upper lobe, bilateral multifocal consolidation, ground-glass opacities, centrilobular nodules, and extensive mucoid impaction of the tracheobronchial tree (Fig. 1). Therapeutic anticoagulation was initiated. During pressure-support ventilation, diaphragmatic ultrasonography showed preserved bilateral diaphragm thickness but a reduced right diaphragmatic thickening fraction of 17% (Figure 2A, B). Serum antibodies against Monosialodihexosylganglioside (GM2), GM3, GM4, and Trisialotetrahexosylganglioside 1a (GT1a) gangliosides were above the laboratory reference thresholds. Serum and cerebrospinal fluid (CSF) paraneoplastic antibody panels were negative. CSF contained 1 nucleated cell/mm^3^, with a glucose level of 3.05 mmol/L and protein level of 0.35 g/L. Microbiologic studies were negative, and cytologic and flow-cytometric examinations showed no malignant cells. Nerve conduction studies showed a generalized sensorimotor neuropathy. Compound muscle action potential amplitudes were reduced in multiple nerves. Motor responses were absent in the right peroneal and tibial nerves, and sensory responses were absent in both sural and superficial peroneal nerves (Table 2). Baseline needle electromyography (EMG) was limited by technical interference. No fibrillation potentials or positive sharp waves were detected in the muscles that could be sampled, but motor unit morphology and recruitment could not be assessed reliably. Bronchoscopy showed abundant purulent secretions throughout both bronchial trees. Direct endobronchial stimulation failed to elicit a cough, consistent with impaired airway protection (Figure 2C, D). Sural nerve biopsy showed moderate loss of myelinated fibers and axonal degeneration. Biceps brachii biopsy showed chronic neurogenic changes, increased major histocompatibility complex class I expression, and complement deposition. These findings were nonspecific and did not establish an immune-mediated neuropathy or myopathy. Myositis-specific antibodies were negative.

**Table 1:** Laboratory findings on admission.

Variable	Value	Reference range
**Arterial Blood Gas (ABG) analysis**		
pH	7.407	7.35–7.45
Partial pressure of carbon dioxide (PaCO_2_), mm Hg	58.60	35–45
Partial pressure of oxygen (PaO_2_), mm Hg	48.90	80–100
Bicarbonate (HCO_3_^−^), mmol/L	36.10	22–26
Lactate, mmol/L	1.30	0.5–1.6
**Complete Blood Count (CBC**)		
White-cell count, ×10^9^/L	6.66	4.0–10.0
Red-cell count, ×10^12^/L	2.88	4.5–5.5
Hemoglobin, g/L	77	130–175
Platelet count, ×10^9^/L	122	150–400
Neutrophils, %	86.6	40–75
Absolute neutrophil count, ×10^9^/L	5.76	2.0–7.0
**Inflammatory Marker**		
High-sensitivity C-reactive protein, mg/L	118.43	<5.0
PCT, mg/L	0.66	<0.05
D-Dimer, mg/L	1.21	<0.24

**Table 2:** Electrophysiologic findings before and after treatment.

Nerve	Measure	Before treatment	After treatment
**Motor nerve conduction studies**			
**Left ulnar**	DML, ms	3.75	3.69
	CMAP, distal/proximal, mV	1.57/1.39	2.24/2.01
	MCV, m/s	47.1	48.3
**Right ulnar**	DML, ms	3.71	3.65
	CMAP, distal/proximal, mV	0.60/0.93	1.35/1.05
	MCV, m/s	44.5	46.2
**Left median**	DML, ms	4.15	4.17
	CMAP, distal/proximal, mV	1.65/1.30	2.10/2.10
	MCV, m/s	39.7	47.1
**Right median**	DML, ms	ND	5.46
	CMAP, distal/proximal, mV	ND	1.04/1.03
	MCV, m/s	ND	33.6
**Left peroneal**	DML, ms	5.77	5.96
	CMAP, distal/proximal, mV	2.00/2.00	3.30/2.70
	MCV, m/s	42.0	45.3
**Right peroneal**	DML, ms	NR	5.98
	CMAP, distal/proximal, mV	NR	0.51/0.50
	MCV, m/s	NR	48.5
**Left tibial**	DML, ms	6.15	6.96
	CMAP, distal/proximal, mV	1.49/1.08	1.42/1.31
	MCV, m/s	41.7	35.2
**Right tibial**	DML, ms	NR	7.99
	CMAP, distal/proximal, mV	NR	0.09/NR
	MCV, m/s	NR	NR
**Sensory nerve conduction studies**			
**Left ulnar**	SNAP/SCV, µV; m/s	4.9/45.7	3.9/45.5
**Right ulnar**	SNAP/SCV, µV; m/s	4.0/48.6	4.3/47.6
**Left median**	SNAP/SCV, µV; m/s	6.5/58.4	7.6/46.2
**Right median**	SNAP/SCV, µV; m/s	ND	1.53/47.3
**Left sural**	SNAP	NR	NR
**Right sural**	SNAP	NR	NR
**Left superficial peroneal**	SNAP	NR	NR
**Right superficial peroneal**	SNAP	NR	NR
**Late responses and repetitive nerve stimulation**			
**Left ulnar**	F-wave minimum latency/persistence	ND	31.3 ms/40%
**Right ulnar**	F-wave minimum latency/persistence	ND	32.9 ms/60%
**Left tibial**	F wave	ND	NR; M latency ~5.7 ms
**Right tibial**	F wave	ND	NR; M latency ~5.9 ms
**Left ulnar**	Repetitive nerve stimulation	No significant decrement	ND
**Needle electromyography**			
**Sampled muscles**	Spontaneous activity and motor unit morphology	Examination technically unsuccessful because of interference; no interpretable data obtained	Chronic neurogenic motor unit changes with reduced recruitment in multiple upper- and lower-extremity muscles; positive sharp waves in selected lower-extremity muscles

Values were transcribed from the original electrodiagnostic reports. CMAP amplitudes are presented as distal/proximal values. The baseline and follow-up examinations did not use identical late-response or needle-electromyography protocols; therefore, changes in F waves, spontaneous activity, motor unit morphology, and recruitment should not be interpreted as direct quantitative comparisons. The reappearance of a previously absent response indicates improved recordability or nerve excitability but does not, by itself, establish axonal regeneration or treatment efficacy.

CMAP = compound muscle action potential, DML = distal motor latency, MCV = motor conduction velocity, ND = not done or not documented in the available records, NR = no response, SCV = sensory conduction velocity, SNAP = sensory nerve action potential.

**Figure 1.**
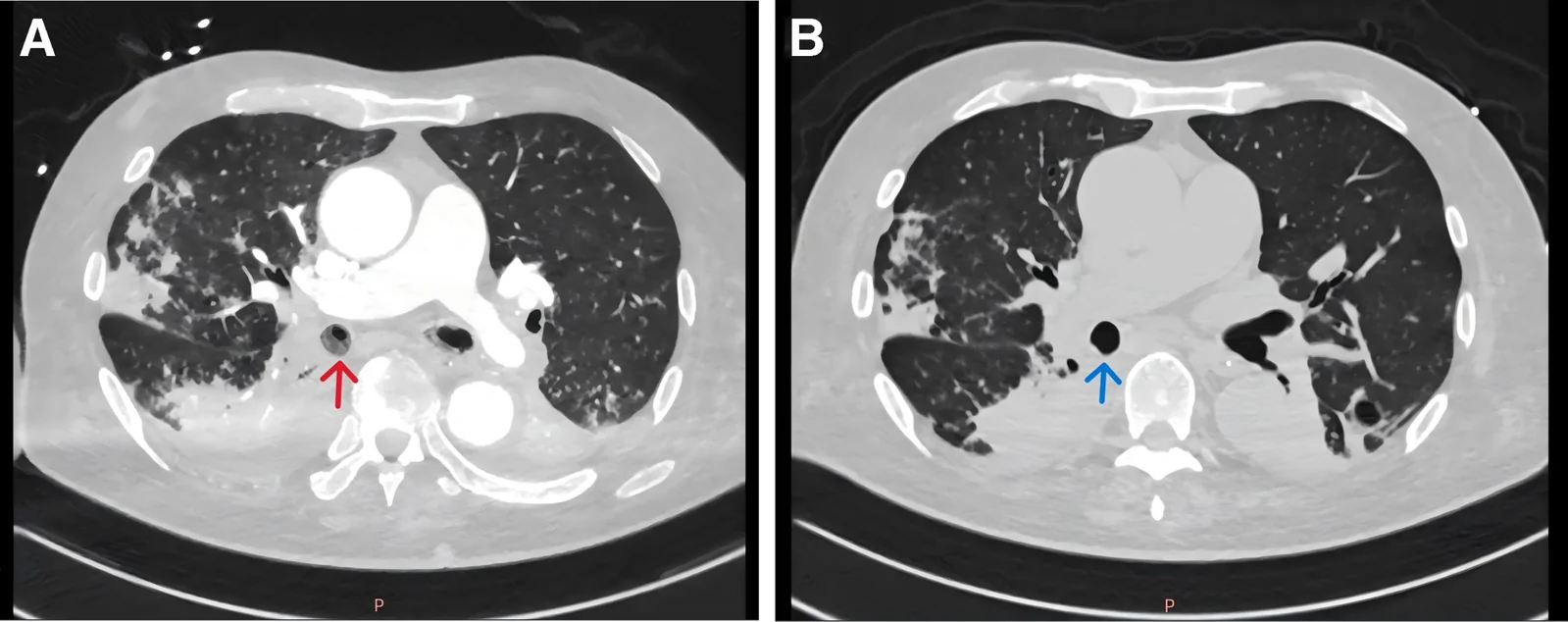
Axial computed tomographic pulmonary angiography images at the same thoracic level. (A) Baseline scan obtained on hospital admission. The red arrow indicates mucoid impaction (retained sputum) within the right main bronchus. Bilateral multifocal pulmonary consolidation and ground-glass opacities are also visible, consistent with concurrent pneumonia. (B) Follow-up scan after anti-infective therapy and aggressive airway clearance. The blue arrow demonstrates complete resolution of the previously identified mucoid impaction at the same site, accompanied by partial absorption of pulmonary parenchymal inflammatory lesions.

**Figure 2.**
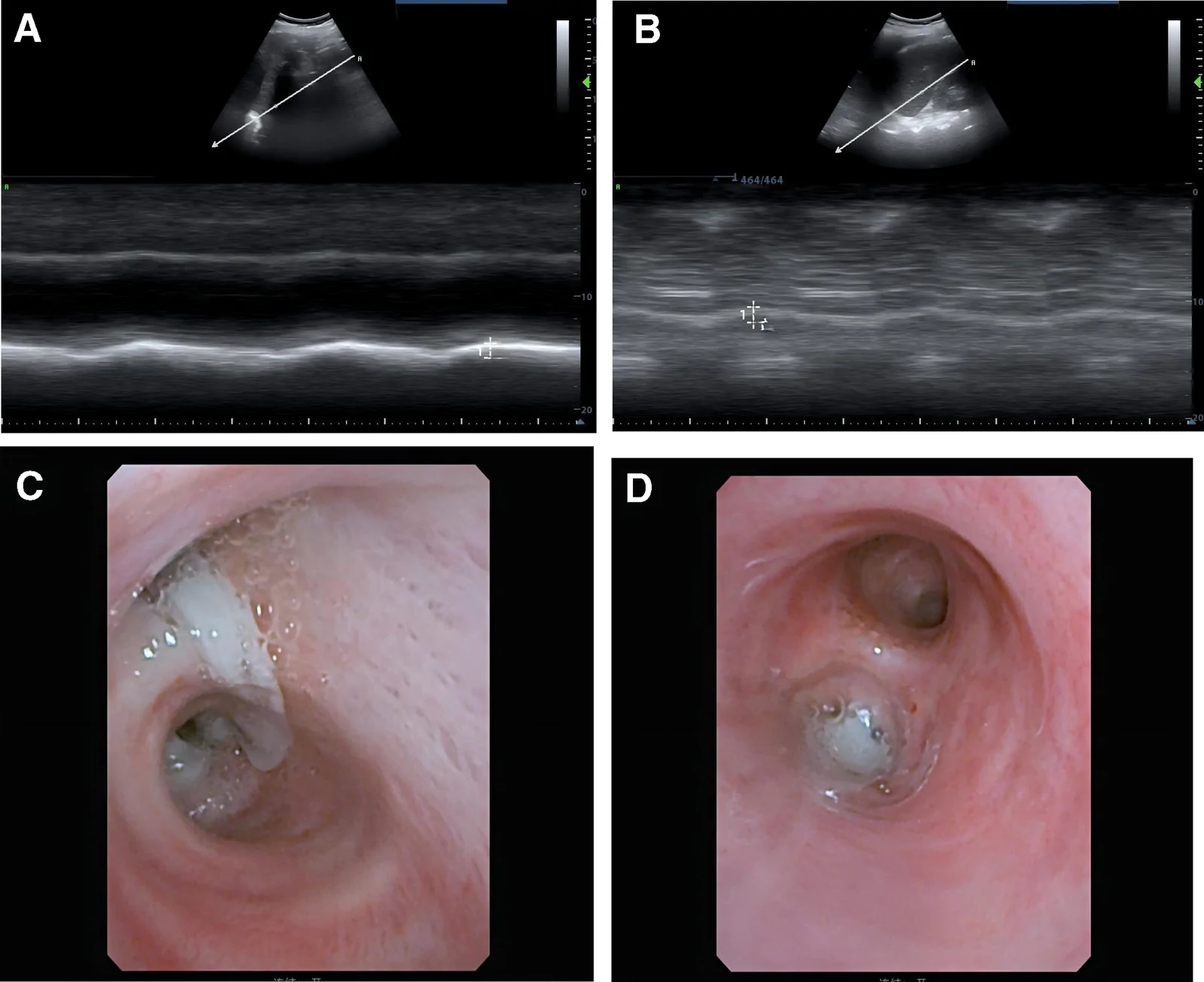
Diaphragmatic ultrasonographic and bronchoscopic findings. (A, B) Diaphragmatic ultrasound images obtained during pressure-support ventilation, showing preserved bilateral diaphragm thickness and a reduced right diaphragmatic thickening fraction of 17%. (C, D) Bronchoscopic images revealing abundant purulent secretions throughout both bronchial trees, with no elicitable cough reflex upon direct endobronchial stimulation.

The respiratory failure was attributed to pneumonia, extensive secretion retention, and pulmonary embolism superimposed on chronic bulbar and respiratory muscle weakness. A superimposed immune-mediated polyradiculoneuropathy was initially considered because of the recent deterioration, generalized areflexia, sensorimotor nerve conduction abnormalities, and anti-ganglioside antibody positivity. However, the longstanding neuromuscular syndrome, normal CSF protein level, and absence of definite electrophysiologic evidence of acquired demyelination limited the certainty of this diagnosis.

On hospital day 1, the patient received pressure-support ventilation (FiO_2_, 0.50; positive end-expiratory pressure, PEEP, 6 cm H_2_O; pressure-support, 12 cm H_2_O). Cefoperazone-sulbactam and ambroxol were administered empirically. After carbapenem-resistant *Pseudomonas aeruginosa* was isolated from sputum, antimicrobial therapy was changed on hospital day 5 to ceftazidime-avibactam (2.5 g every 8 hours). Airway clearance included postural drainage, daily chest percussion and mechanical vibration, prone positioning, and repeated bronchoscopic suctioning.

Because a superimposed immune-mediated polyradiculoneuropathy remained a consideration, therapeutic plasma exchange was performed on hospital days 9, 12, 14, and 16. The records documented administration of 2000 mL of plasma per session.

During treatment, inflammatory markers decreased and FiO_2_ was reduced to 0.30. By hospital day 14, muscle strength had increased from Medical Research Council grades 0 to 1 at admission to grades 3 to 4+ in the upper extremities and grade 3 in the lower extremities. These changes occurred during concurrent treatment of infection, respiratory support, airway clearance, and plasma exchange and could not be attributed to a single intervention.

After completing a spontaneous breathing trial, the patient was extubated on hospital day 16 and transitioned to high-flow nasal oxygen (FiO_2_, 0.35; flow, 30 L/minutes). A cough response became clinically detectable, although vital capacity, respiratory pressures, and peak cough flow were not measured. On hospital day 18, secretion retention recurred, with an oxygen saturation of 85% and severe hypercapnic respiratory failure (pH, 7.160; PaO_2_, 60 mm Hg; PaCO_2_, 98.8 mm Hg; bicarbonate, 33.8 mmol/L). Suctioning provided only transient improvement, and the patient was reintubated. Synchronized intermittent mandatory ventilation was initiated (FiO_2_, 0.35; positive end-expiratory pressure, 8 cm H_2_O; pressure-support, 10 cm H_2_O; tidal volume, 350 mL). Extubation failure was attributed to persistent secretion retention, impaired airway protection, and limited ventilatory reserve. Antibiotics were discontinued on hospital day 18 after clinical improvement and a decrease in inflammatory markers.

Rehabilitation began on hospital day 19 and included occupational therapy for physical dysfunction, pulmonary rehabilitation, coordination and transfer training, balance exercises, endurance training, and proprioceptive neuromuscular facilitation.

Follow-up nerve conduction studies showed higher compound muscle action potential amplitudes in several nerves and a recordable right peroneal motor response that had been absent at baseline. Sensory responses remained absent in both sural and superficial peroneal nerves. Because the examinations were performed over a short interval under different technical conditions, these changes were not considered evidence of axonal regeneration or treatment response. Follow-up needle EMG showed widespread chronic neurogenic motor unit changes, reduced recruitment, and active denervation in selected lower-extremity muscles, supporting a chronic lower motor neuron disorder.

Genetic testing identified an expanded CAG repeat in exon 1 of the androgen receptor gene, confirming SBMA. This diagnosis accounted for the longstanding progressive weakness, bulbar dysfunction, muscle atrophy, areflexia, and sensory nerve conduction abnormalities. Leuprolide acetate was administered as a single subcutaneous injection at a dose of 3.75 mg for androgen suppression during hospitalization.

Because of persistent secretion retention, impaired airway protection, and the risk of recurrent extubation failure, tracheostomy was performed on hospital day 26. Bronchoscopic suctioning, humidified oxygen through the tracheostomy, airway clearance therapy, and rehabilitation were continued. The patient was weaned from invasive ventilation and transferred to the general ward on hospital day 30. He was discharged to a rehabilitation hospital on hospital day 44. The summary of all medical histories and treatment processes is shown in Figure 3.

**Figure 3.**
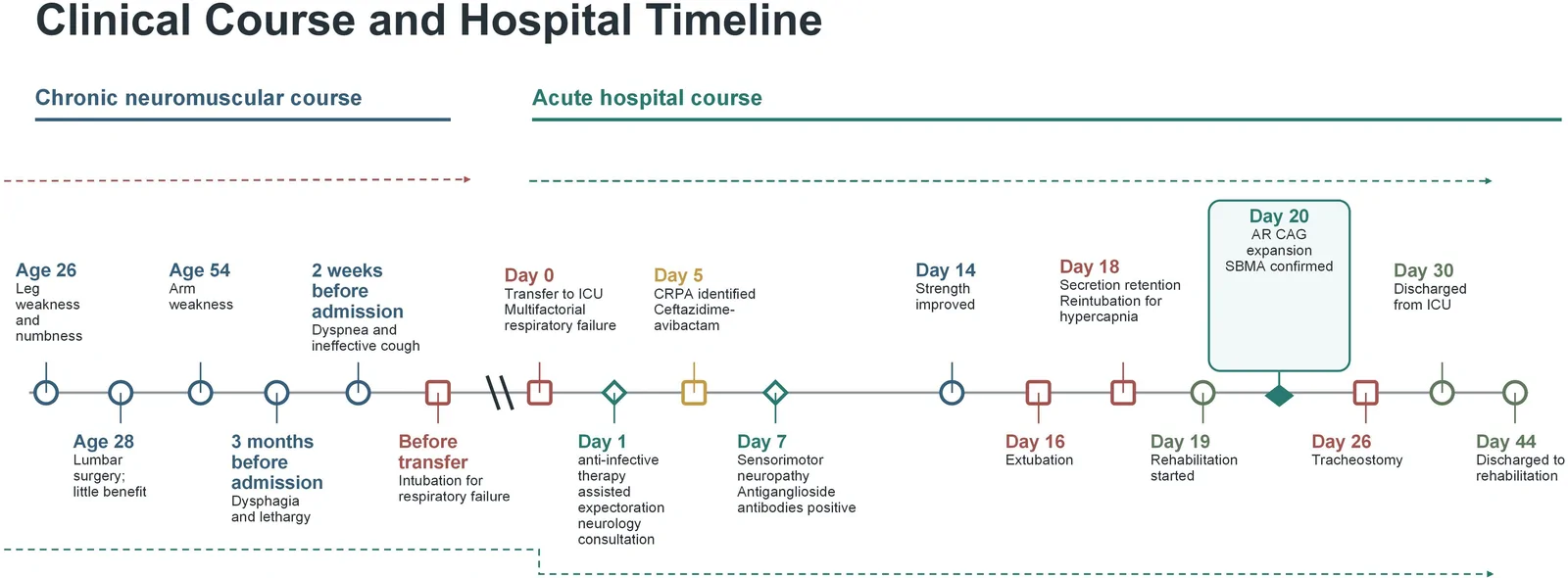
Timeline of the patient’s clinical course and key interventions during hospitalization. The schematic summarizes disease progression, diagnostic workup, therapeutic strategies, and clinical outcomes from admission to discharge to a rehabilitation facility.

## 3. Discussion and conclusions

Kennedy’s disease is a rare X-linked progressive neurodegenerative disorder.^[1]^ The clinical manifestations include hand tremors, limb weakness, gynecomastia, orofacial fasciculation, dysarthria, and dysphagia. Although respiratory involvement is uncommon, it represents the primary cause of mortality in affected individuals.^[6]^

The acute respiratory failure in this patient was likely multifactorial rather than attributable to a single neuromuscular process. Pneumonia and pulmonary embolism could account for the abrupt respiratory deterioration and increased ventilatory demand, while ineffective cough, dysphagia, and respiratory muscle weakness from previously unrecognized SBMA probably reduced pulmonary reserve and impaired secretion clearance. However, neither pneumonia nor pulmonary embolism adequately explains the decades-long progression from lower- to upper-extremity weakness followed by bulbar symptoms. ICU-acquired weakness was also an important competing or superimposed diagnosis because infection, immobilization, and mechanical ventilation can produce critical illness polyneuropathy, critical illness myopathy, or both, including respiratory muscle weakness and difficulty liberating patients from ventilatory support.^[8,9]^ Nevertheless, the substantial neuromuscular symptoms preceding critical illness argue against ICU-acquired weakness as the primary disorder. Taken together, the chronic phenotype and pathogenic AR CAG expansion support genetically confirmed SBMA with acute decompensation precipitated or amplified by cardiopulmonary disease and critical illness.

In a 20-year follow-up study of 223 Japanese patients with Kennedy’s disease, the most common cause of death was pneumonia secondary to aspiration and dysphagia.^[7]^ Spirometric measurements can assess respiratory function, specifically pulmonary ventilation and gas exchange, enabling the detection of early-stage breathing impairments. A longitudinal study demonstrated a progressive decline in maximum inspiratory pressure (MIP), maximum expiratory pressure (MEP), and forced vital capacity (FVC) over an 11-year observation period in SBMA patients. Specifically, the average declines from the first year to the eleventh year of observation were 25%, 42%, and 52% for FVC, MIP, and MEP, respectively.^[10]^ The greater decrease in the MEP and MIP values than in the FVC value suggests that respiratory muscle strength decreases earlier and more significantly. A longitudinal study further demonstrated correlations between motor function and both forced vital capacity (FVC) and peak expiratory flow.^[11]^ PEF is the maximum instantaneous flow rate generated during a forced expiration and serves as an indicator of how quickly a subject can exhale, providing a reliable global assessment of voluntary cough efficacy in mitigating the risk of aspiration pneumonia.^[12]^ The observed reduction in the inspiratory thickening fraction of the diaphragm also indicates dysfunction of the respiratory muscles. This clinical course corroborates recurrent aspiration pneumonitis due to impaired airway clearance, thereby necessitating daily bronchoscopic interventions for management. Therefore, it is essential to emphasize the importance of respiratory muscle strength in the early stages and implement comprehensive management of swallowing and respiratory function for the long-term care of patients with SBMA. Conducting annual pulmonary function tests and engaging in swallowing rehabilitation exercises represent prudent clinical practices.^[13]^

Beyond the chronic respiratory decline attributable to SBMA, the patient experienced rapid neurological deterioration within 2 weeks following a respiratory tract infection, characterized by worsening limb weakness and diaphragmatic dysfunction. The acute, monophasic onset of bilateral limb weakness, reduced or absent deep-tendon reflexes, and progression to nadir within 28 days was consistent with the core clinical features of GBS according to the Brighton Collaboration criteria.^[14]^ Nerve conduction studies demonstrated a generalized sensorimotor neuropathy, with reduced CMAP amplitudes in multiple nerves, absent right peroneal and tibial motor responses, and absent bilateral sural and superficial peroneal sensory responses (Table 2); baseline needle EMG was uninterpretable due to technical interference, precluding longitudinal assessment of denervation and reinnervation. Serum anti-ganglioside antibody positivity (GM2, GM3, GM4, and GT1a), combined with pathological findings from the right sural nerve and right biceps brachii muscle, further supported suspicion of an immune-mediated peripheral neuropathy. CSF analysis was unremarkable without albumin-cytologic dissociation; although this finding was not supportive of GBS, it did not rule out the diagnosis. Diagnostic attribution was complicated by the patient’s genetically confirmed SBMA, which could account for chronic neurogenic and sensory abnormalities, but the acute progression to nadir: including respiratory involvement: clearly distinguished this episode from the typically chronic course of SBMA.^[15]^ GBS is characterized by immune-mediated attack on myelin components, leading to demyelination and secondary axonal damage, and is typically categorized into acute inflammatory demyelinating polyradiculoneuropathy and acute motor axonal neuropathy based on target antigen differences.^[15]^ The acute motor axonal neuropathy subtype, which typically presents with primary axonal injury without substantial T-cell inflammation or demyelination, was consistent with the right sural nerve biopsy results in this case. As reported by Alex Y. Doets et al the axonal subtype accounts for a higher proportion of GBS cases in Bangladesh (36%) than in Europe/Americas (6%) and other Asian countries (6%)^[16]^; patients with axonal GBS rarely show sensory impairment, and those with pharyngeal–cervical–brachial weakness are more likely to test positive for IgG anti-GT1a antibodies.^[17]^ Four cycles of therapeutic plasma exchange were administered, and the subsequent partial symptomatic improvement and modest electrophysiologic changes (Table 2) offered only limited supportive evidence for the hypothesis that immune-mediated pathology underlay the recent clinical decline. Taken together, we hypothesize that this patient presented with GBS superimposed on underlying SBMA. To our knowledge, the co-occurrence of SBMA and probable GBS has not been documented previously, and acute deterioration in patients with SBMA should therefore trigger evaluation for a superimposed, potentially treatable neuromuscular disorder.

This case report has several inherent limitations. Most importantly, the diagnosis of superimposed GBS remains only probable, not definitive: the absence of CSF albuminocytologic dissociation, technically constrained baseline needle EMG, nonspecific nerve and muscle biopsy findings, and confounding chronic neurogenic injury from underlying SBMA collectively prevent a confirmed diagnosis of immune-mediated polyradiculoneuropathy, and alternative causes of acute neurological deterioration such as critical illness polyneuropathy cannot be completely ruled out. Additionally, as a single-case descriptive study, the findings have limited generalizability; clinical improvement occurred during concurrent multidisciplinary interventions including anti-infective therapy, respiratory support, airway clearance and rehabilitation, so the independent therapeutic contribution of plasma exchange cannot be isolated. Incomplete procedural parameters of plasma exchange and lack of long-term follow-up further restrict the robustness of the conclusions.

In summary, reports on the intensive care unit (ICU) management of Kennedy’s disease remain exceedingly limited. We report a case of genetically confirmed SBMA with superimposed probable GBS, which was successfully managed via a multidisciplinary strategy including respiratory support, anti-infective therapy, bronchoscopic sputum clearance, respiratory rehabilitation, and therapeutic plasma exchange. This case underscores the clinical value of integrated multidisciplinary care for acute decompensation in severe SBMA, and provides practical reference for the management of similar clinical scenarios.

## Acknowledgments

We are grateful to the patient and his legally authorized representative for consenting to this report, and we acknowledge the clinical staff for their expert support.

## Author contributions

**Data curation:** Xinran Gao.

**Investigation:** Xinran Gao.

**Supervision:** Kunyao Yu.

**Writing – original draft:** Xinran Gao.

**Writing – review & editing:** Kunyao Yu.
